# Soil Bacteria in Archaeology: What Could Rank Abundance Functions Tell Us About Ancient Human Impacts on Microbial Communities?

**DOI:** 10.3390/microorganisms12112243

**Published:** 2024-11-06

**Authors:** J. Michael Köhler, Linda Ehrhardt, P. Mike Günther, Jialan Cao

**Affiliations:** Institute for Micro- and Nanotechnologies/Institute for Chemistry and Biotechnology, Technische University Ilmenau, PF 10 05 65, D-98684 Ilmenau, Germany; linda.ehrhardt@tu-ilmenau.de (L.E.); mike.guenther@tu-ilmenau.de (P.M.G.); jialan.cao@tu-ilmenau.de (J.C.)

**Keywords:** bacterial communities, soil, archaeology, rank order functions, modelling, human impact

## Abstract

Metagenomic analysis of soil bacterial communities based on 16S rRNA reflects a typical community composition containing a low number of high-abundance types and a very high number of low-abundance types. Here, the formation of characteristic rank order functions of bacterial abundance is investigated by modelling the dynamics of soil bacterial communities, assuming a succession of different bacterial populations that grow rapidly and decay more slowly. We found that the characteristic shape of typical rank order functions is well reflected by simulations. In addition, our model allowed us to investigate strong disturbances in the soil, which could be expected in cases of strongly changing local environmental conditions in soil, e.g., after translocation and covering of soil material. Such events could lead to the formation of shoulders in the rank order functions. Abundance rank orders observed in cases of some archaeological soil samples do indeed show such a shoulder and could be well interpreted by simulated rank order functions. As a result, it can be concluded that the investigations herein support our hypothesis that abundance rank orders contain information about the temporal order of developing bacterial types in changing communities and thus store information about local environmental conditions in the past, including ancient humans’ impact on soil. This information can be used for interpretation of archeological findings and for reconstruction of different former human activities, as well as knowledge on the translocation of soil material in the past.

## 1. Introduction

The composition of soil microbial communities is typically characterized by a mixture of highly abundant and low-abundance types [1]. The character of the community depends on physical environmental parameters such as temperature and humidity but is also strongly dependent on chemical parameters such as soil pH, salinity, organic nutrients, and bioavailability of trace elements. In addition, ecological interactions between macro- and microorganisms are an important factor. This includes promotion or suppression by secondary metabolites and metabolic couplings among soil bacteria [2].

Soils are composed mostly of a complex spatial structure consisting of a hierarchy of inorganic and organic particles with sizes between millimetres and nanometers and a corresponding pore size hierarchy. This results in a certain decoupling of small subspaces in which different compositions of microbial communities can evolve in a close neighbourhood. The high microbial diversity found in many soils is related to this three-dimensional network of small local biocenoses, which are more or less weakly connected by the—mostly diffusive—exchange of chemical components in the pore water and the active motion of microorganisms.

The composition of a soil bacterial community has to be understood through its dynamic behaviour [3]. It has to be assumed that in each local microbial community, only a few types are highly metabolically active, according to the specific local growth conditions. These fast-growing types dominate the bacterial community in terms of abundance. Apart from these types, we must expect other slower-growing types and non-growing types with a low level of metabolic activity. In addition, a huge number of low- and very-low-abundance types without metabolic activity are to be expected, representing so-called “dormant” states [4]. The large number of different low-abundance bacteria which form the high diversity in soil are responsible for the flexibility in the reactions of soil microbial communities to environmental changes. The ensemble of dormant types preserves information about earlier existing different environmental conditions [5,6,7]. On the other hand, it forms a reservoir for responding to possible future environmental changes [8,9]. As detailed above, this picture of the expected functions and structure of local soil bacterial communities results in a typical form of rank abundance distribution (RAD). 

RADs have been used for many decades to quantitatively describe the composition of communities. Typically, they are applied for ordering related species. The abundance functions are marked by a non-linear character, always reflecting the mixture of a few high-abundance types with a moderate number of medium-abundance types and a large number of low-abundance species. Different models have been developed to describe RADs, including log-normal distributions [10,11,12,13,14]. These quantitative mixtures should be understood not only as an expression of recent ecological relationships and niche differentiation but also as a result of a certain ecological evolution [15]. The community reflects both recent local environmental conditions and their previous changes in that locale [16,17]. This view is consistent with the concept of “ecological memory”, in which each community has to be regarded as a product of the local environmental history of a place [18]. The effect of ecological memory is relevant in short timescales, where highly dynamic bacterial communities evolve, but it also affects slow processes and larger timescales [19]. The composition of soil microbial communities and the related memory effects are important for soil fertility, agriculture, and forest management [20].

Alongside naturally occurring environmental changes, the impact of human activities on soil modifies the growth behaviour and competitive conditions of soil bacteria. Recent evidence suggests that the components of soil bacterial communities can supply information about changes in soil due to the former use of places by humans [21,22,23]. This human impact is quite obvious in recent changes due to industrial activities or the release of contaminations into the environment [24,25,26,27] but also seems to play a role through pre-industrial handcraft, settlement, mining, and agricultural activities that may date back to prehistory [28,29,30,31,32].

Here, it is hypothesized that specific former human impacts led to considerable changes in the local composition of soil bacterial communities in the past and that the translocation of soil material, the covering of former surfaces by younger soil material deposits, and the burying of a soil layer caused a certain kind of conservation of specific information regarding human-impacted bacterial communities. Thus, this former local ecological situation has been preserved to some extent and can be read out through NGS analyses.

In the following, datasets of 16S rRNA sequences from soil samples from different places, including soil samples from archaeological excavations, are used to search for possible traces of ancient humans’ impact on the abundance distributions of soil bacteria. Therefore, in particular, the rank orders of bacterial abundance are used to obtain simple comparable pictures of differences in the quantitative compositions of bacterial communities in buried soils.

## 2. Experimental Procedure

### 2.1. Sample Set

The examples of soil samples for this study originated from special places marked by different ancient human impacts. The selection of samples used includes surface soil samples from pre-industrial mining areas and samples of buried soil from archaeological investigations of pre-industrial saline activity and prehistoric burial and settlement sites. Table 1 provides an overview of the soil samples used.

### 2.2. Sequencing and Data Analysis

DNA of soil samples was extracted, and the 16S rRNA-related sequence was amplified by PCR following standard procedures. The whole process of sample preparation, selective amplification, sequencing, and data analysis was described previously [33].

Empirical rank order functions were obtained by ordering the proven OTUs by the obtained numbers of reads from the 16S rRNA section of genomic DNA.

## 3. Results and Discussion

### 3.1. Temporal Order-Related Hypothesis for the Interpretation of Rank Orders in Bacterial Abundances

Regarding the growth rate of bacteria, at least two different cases have to be distinguished. On the one hand, there are bacteria which grow and multiply rapidly under optimal conditions but grow slowly or stagnate under less suitable environmental conditions. On the other hand, there are bacteria that always grow slowly or stagnate. For the latter group, it is difficult to determine from NGS data whether the bacteria are active or inactive if their abundances are low compared to the abundances of other bacteria at the same place. It has to be assumed that such bacteria are usually present only in low concentrations. 

In contrast, fast-growing bacteria can be high- or low-abundance, with high abundance always signalling recent high growth activity or high activity in the recent past. If the local environmental conditions—nutrient availability and humidity, among others—are changed, a fast-growing bacterial type cannot grow any longer. Vegetative cells of this bacterium may die quickly. However, a certain part of the cells might switch into a dormant state, like spores in the case of spore-forming species. In this state, the bacterium can survive for a long time, in principle. Meanwhile, there is evidence that such dormant cells can survive and remain capable of reproduction for millennia or even millions of years under suitable conditions. It can be assumed that the concentration of dormant cells of a formerly active bacterium also decreases, but much more gradually than in the case of vegetative cells. This would result in a decaying function with kinetic parameters depending on the sensitivity of the bacterium and the aggressiveness of the changed environment. Despite these individual differences, a certain tendency can be expected, that is, that bacteria that had been active for a long time will be decayed to lower concentrations than bacteria with formerly active phases in the recent past. As a result, the rank orders of bacterial abundance include, to a certain extent, a temporal order of the former activity of bacterial types. 

**Figure 1:**
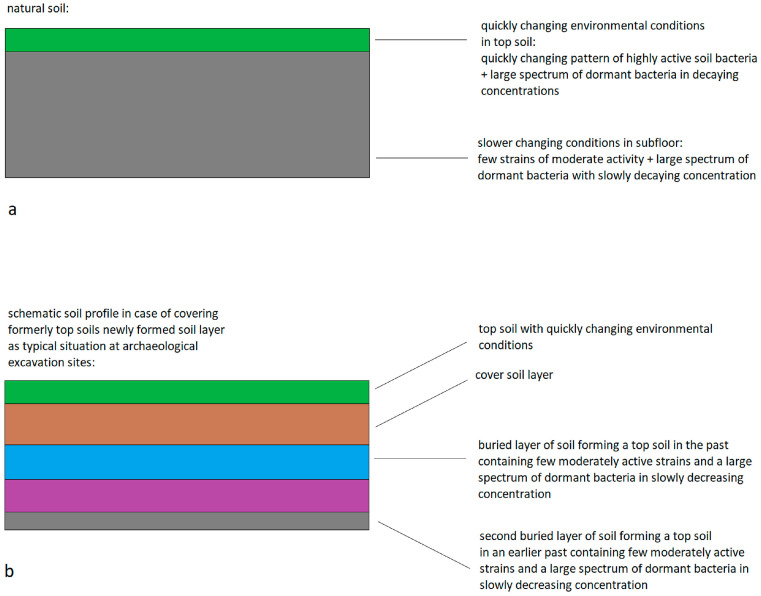
Soil profiles (schematically) (**a**) in the case of natural—widely undisturbed—layers and (**b**) for an example of human-impacted soil (including translocation of soil material and burial of former surface material) and soil containing deposits connected with ancient human activities.

It has to be assumed that the decay functions are different between the topsoil layer and the bottom soil layer. It must be considered that the faster and stronger changing environmental conditions in the topsoil layer cause a rapid change in the patterns of highly active bacterial strains. The decay of less active and dormant bacteria is probably slower in deeper layers of undisturbed soil. When formerly active topsoil layers are buried by natural or human activities, the topsoil layers and their bacterial communities are brought into the conditions of ground soil. On the one hand, the chemical conditions of the soil can be stored and cause a certain composition of the bacterial community in the buried soil. On the other hand, the concentrations of former types of active and less active bacteria are slowly decreasing (Figure 1). Therefore, it can be expected that bacterial community patterns of soil samples from archaeological excavations not only store information about the preserved specific chemical conditions in the soil (e.g., the composition of nutrients, pH, salinity, toxic components) but also carry information about non-preserved former soil conditions corresponding to less abundant decaying components of former bacterial communities.

### 3.2. Model of Formation of Rank Order Functions by Successive Changes in Growth Conditions

In this section, a very simple model is proposed to quantitatively describe the formation of a rank order function of soil bacterial communities. This model is based on the assumption that the rank order function is determined, at least partially, by a succession of dominant bacteria due to changing environmental conditions. It is clear that the environmental changes occur on very different timescales and include periodic changes, such as seasonal conditions. Thus, the resulting abundances have to be understood as a complex superposition of the responses of all bacteria to changes on different timescales. Despite this complexity, a highly simplified approach is proposed below to develop a way to illustrate the possible formation of rank order functions of bacterial abundances which is mainly determined by the temporal succession of bacterial activities.

Quick changes in local environmental conditions can occur by natural processes, for example by flooding, sudden erosion events, or earthquakes. However, they can also be caused by human activities such as building of houses and stables, concentration of animals for livestock breeding, by traffic, by soil contaminations due to crafts or metallurgy, by exploitation of mines, deposition and translocation of soil material, and by covering of top soils by new soil layers, for example. 

First, it is postulated that the transition between a dormant and a highly active state of a bacterial type occurs quickly. This assumption is based on the fact that exponential growth of the newly promoted bacterial type is most likely after a change in conditions. After exponential growth, the concentration of active bacteria will pass through a more or less extended maximum and then decline. 

**Figure 2:**
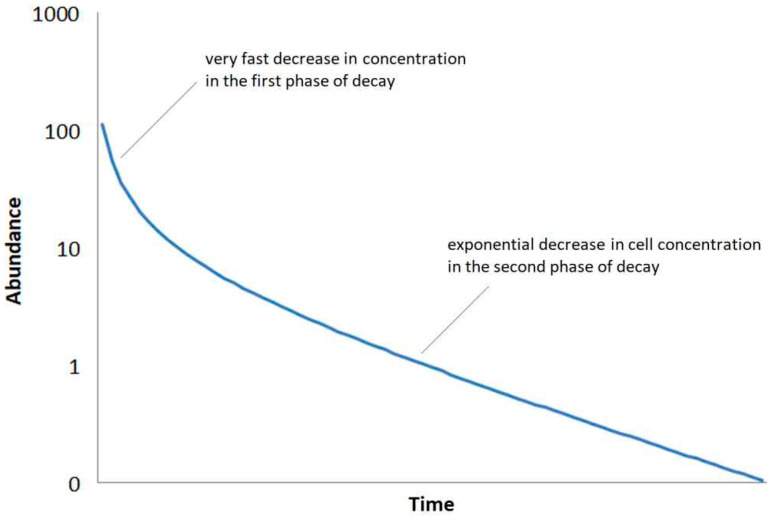
Assumed decay of abundance of a bacterial soil type (density of cells) after a phase of high activity following the decay function of Equation (1).

Second, it is assumed that the decay after a phase of high activity is divided into two phases. In the first phase, a fast decrease in the concentration of bacteria associated with the death of vegetative cells. The second phase is determined by the gradual decrease in the concentration of the dormant forms of cells. This decrease is much slower than the decrease in the first phase (Figure 2). This behavior can be easily simulated by following the iterative decay function:

c_i_ = c_(i−1)_ − [(k_1_ ∗ c_(i−1)_^a^) + (k_2_ ∗ c_(i−1)_)] (1)

The cell concentration for each step c_i_ are given by the cell concentration in the previous step c_(i−1)_ and the rate constants for the both decay reactions k_1_ and k_2_. The strong decay in the first process is simulated by the exponent a (in the case shown in Figure 2, a = 1.8).

**Figure 3:**
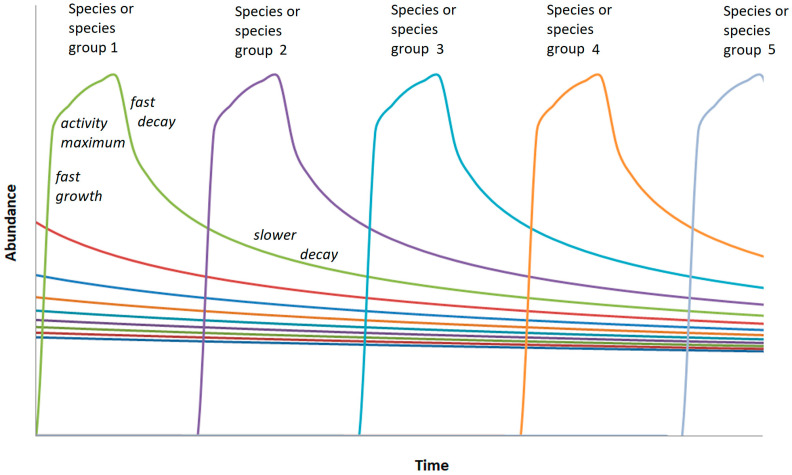
Scheme of succession of dominant OTUs or small groups of them marked by fast growth, a comparatively short phase of high activity (dominance in the soil bacterial community), and decay following a decay function as described by Equation (1).

Third, it is assumed that the abundance rank function results from a succession of dominant bacteria. For the simulation, a simple regular succession is assumed (as shown in the scheme in Figure 3). The resulting abundance distribution then results from the superposition of the successively active bacteria.

Following the simple kinetic rules described above for the single bacteria types, a general rank order function is obtained. This general rank order function can also be described by the superposition of two decay functions. The number of reads N as a function of inverse rank i (ordered by decreasing number of reads) can be described in dependence on the two decay rate constants f_1_ and f_2_ with two additional fitting parameters adapted to the order of magnitude of the highest read number N_max_ and an extrapolated starting read number N_0_ when assuming a simple exponential decay, considering only OTUs with lower and intermediate read numbers:

lg(N) = lg(N_max_/N_0_) ∗ e^−f1∗i^ + [lg(N_0_) − f_2_ ∗ i] (2)

**Figure 4:**
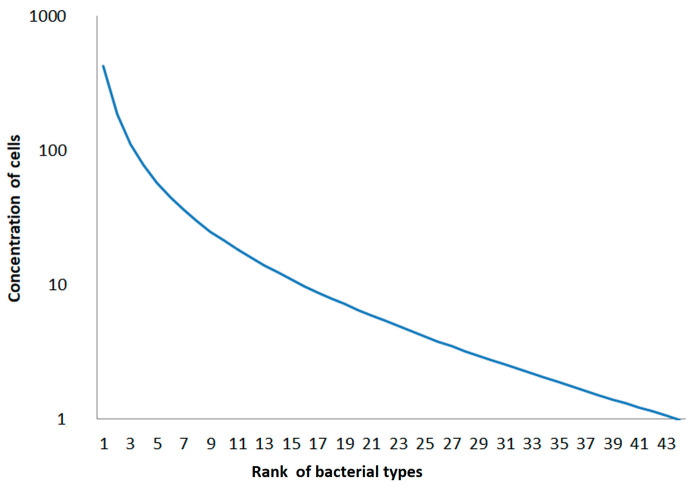
Scheme of a simplified general decay function in abundances of OTUs in soil samples (following Equation (2)); the graph was obtained by superposition of simulated decay of densities of cells following the scheme shown in Figure 3.

In a logarithmic plot, the graph of this function shows a typical shape with a nonlinear decay for the OTUs with the highest abundances (i.e., the lowest inverse rank) and a nearly linear decay for the OTUs with medium and low abundances (i.e., the highest inverse rank, as shown in the scheme in Figure 4). 

The character of this model-derived rank order corresponds rather well with the typical general character of simple rank order functions experimentally found in many soil samples. It reflects the expected character of the abundance distribution with a low number of high-abundance types and a high number of low-abundance bacteria. It has to be emphasized, here, that the proposed two-phase model is a simple empirical approach. It is attractive because a combination of the fast decay of concentrations of vegetative cells after fast-changing environmental conditions and a slow decay of dormant forms is plausible. However, this simple assumption cannot reconsider the whole complexity of metabolic and other ecological relations and their dynamics in soil ecosystems. Thus, this model has to be regarded as a strongly simplified approach, whereas the identification of ecological functions of single species or genera and their changes demands more detailed investigations. 

### 3.3. Comparison of Simulated Rank Order Functions with Abundance Functions of Selected Human-Impacted Places

**Figure 5:**
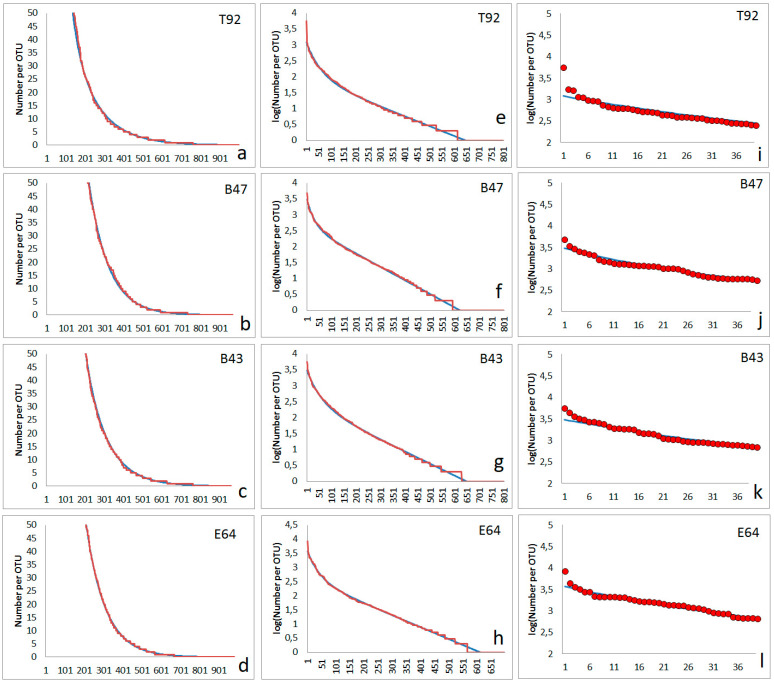
Rank order functions without shoulders obtained from four different soil samples: red lines and circles are experimental abundance data obtained from NGS, and blue lines are fits obtained from the assumed model. First column (**a**–**d**): rank function (number of reads) for lower-abundance OTUs, second column (**e**–**h**): logarithm of abundances for all OTUs), third column (**i**–**l**): abundances (number of reads) for highest-abundance OTUs of each sample. First line (**a**,**e**,**i**): sample T92 (parameters for simulation following Equation (2): N_max_= 1200, N_0_= 96, f_1_ = 0.003075, f_2_ = 0.016); second line (**b**,**f**,**j**): sample B47 (parameters for simulation following Equation (2): N_max_ = 3000, N_0_ = 420, f_1_ = 0.00423, f_2_ = 0.03); third line (**c**,**g**,**k**): sample B43 (parameters for simulation following Equation (2): N_max_ = 3000, N_0_ = 268, f_1_ = 0.00375, f_2_ = 0.016); fourth line (**d**,**h**,**l**): sample E64 (parameters for simulation following Equation (2): N_max_ = 3700, N_0_ = 375, f_1_ = 0.00421, f_2_ = 0.022).

The character of many rank functions observed in the soil bacterial communities can be approximated quite well by the simple model proposed above. Four examples of approximations of experimentally found rank functions are shown in Figure 5. The abundances of an overwhelming number of bacterial types fit satisfactorily with the two-step model (Figure 5a–d). Several hundred OTUs (bacterial types) follow an abundance distribution matching the two-step model (Figure 5e–h). However, the few types with the highest abundances appear to have higher abundances than expected (Figure 5i–l). The four soil samples were taken from the surface. Two of them (T92, B43) originated from the forest soil, one from an agricultural area (B47), and one from the surface of a pre-industrial copper mine deposit (E64).

The interpretation of the fitting parameters has to be performed carefully. One must bear in mind that the values of obtained constants f_1_ and f_2_ are directly dependent on the number of observed OTUs in total in one sample. In cases of high diversity (many OTUs), these constants are lower, and they increase with decreasing number of totally observed OTUs. More remarkable is the fact that the fitting of the read numbers of the overwhelming majority of OTUs is nearly non-affected by the appearance of one or a few OTUs with very high abundances (i.e., the lowest inverse ranks, as visible, for example, for the first dots in Figure 5i–l).

**Figure 6:**
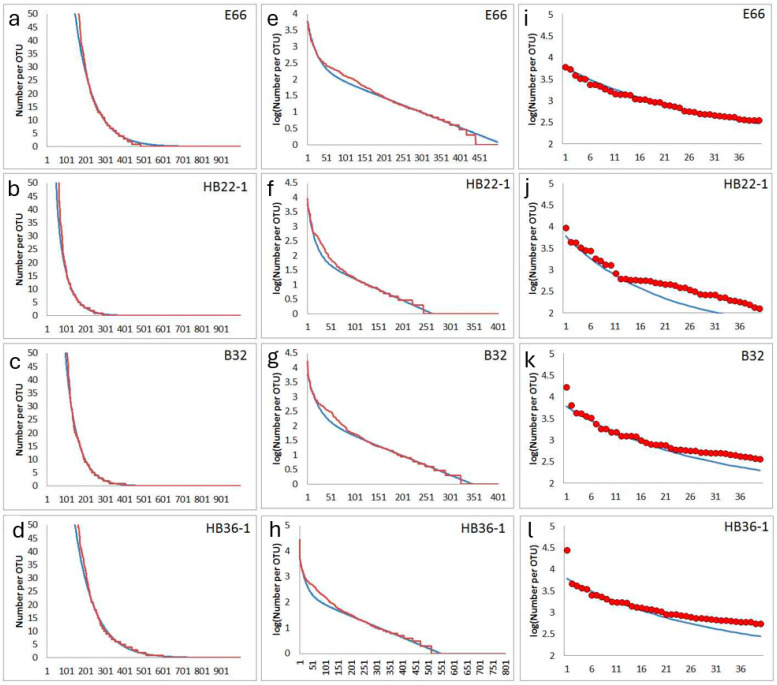
Experimentally observed deviations from the simplified general rank function by a shoulder at medium abundance: red lines and circles are experimental abundance data obtained from NGS, and blue lines are fits obtained from the assumed model. First column (**a**–**d**): rank function (number of reads) for lower-abundance OTUs, second column (**e**–**h**): logarithm of abundances for all OTUs), third column (**i**–**l**): abundances (number of reads) for highest-abundance OTUs of each sample. First line (**a**,**e**,**i**): sample E66 (parameters for simulation following Equation (2): N_max_ = 6000, N_0_ = 227, f_1_ = 0.00457, f_2_ = 0.040); second line (**b**,**f**,**j**): sample HB22-1 (parameters for simulation following Equation (2): N_max_ = 6000, N_0_ = 85, f_1_ = 0.00732, f_2_ = 0.06); third line (**c**,**g**,**k**): sample B32 (parameters for simulation following Equation (2): N_max_ = 6000, N_0_ = 268, f_1_ = 0.00662, f_2_ = 0.045); fourth line (**d**,**h**,**l**): sample HB36-1 (parameters for simulation following Equation (2): N_max_ = 6000, N_0_ = 199, f_1_ = 0.00419, f_2_ = 0.040).

In some cases, deviations from the simple general rank order function were observed concerning a larger group of medium-abundance OTUs. A typical phenomenon is the formation of a weak shoulder (Figure 6). It was supposed that these deviations may be related to perturbations of soil bacterial communities, such as translocation of soil material and its burial by deposition of cover layers [33]. Such an event can mean a rapid and drastic change in local environmental conditions. Such a disturbance could cause a strong reduction of all contained bacteria and the development of new types. Two of the samples come from archaeological excavations, one (HB22-1) from the direct environment of a recovered prehistorical bronze ring and the other one (HB36-1) from excavation of a pre-industrial tannery area [19]. The other both samples come from surface soil, one from a pre-industrial copper mining area (E66) and the other (B32) from a forest.

**Figure 7:**
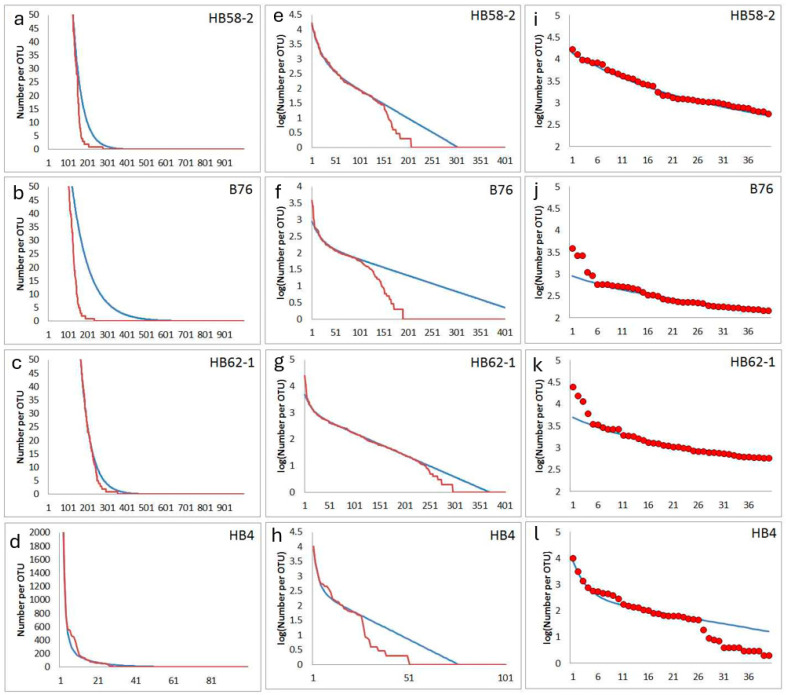
Experimentally observed deep steps from high to medium and low abundances in the rank function: red lines and circles are experimental abundance data obtained from NGS, and blue lines are fits obtained from the assumed model. First column (**a**–**d**): rank function (number of reads) for lower-abundance OTUs, second column (**e**–**h**): logarithm of abundances for all OTUs), third column (**i**–**l**): abundances (number of reads) for highest-abundance OTUs of each sample; first line (**a**,**e**,**i**): sample HB58-2 (parameters for simulation following Equation (2): N_max_ = 14,000, N_0_ = 785, f_1_ = 0.00952, f_2_ = 0.048); second line (**b**,**f**,**j**): sample B76 (parameters for simulation following Equation (2): N_max_ = 900, N_0_ = 192, f_1_ = 0.00481, f_2_ = 0.050); third line (**c**,**g**,**k**): sample HB62-1 (parameters for simulation following Equation (2): N_max_ = 5000, N_0_ = 1136, f_1_ = 0.00826, f_2_ = 0.070); fourth line (**d**,**h**,**l**): sample HB4 (parameters for simulation following Equation (2): N_max_ = 8000, N_0_ = 311, f_1_ = 0.03286, f_2_ = 0.35).

Some samples supplied rank orders in which a massive step in the abundance distribution was observed (Figure 7). Such plots seem to reflect the missing of many medium and low-abundance types. It is possible that a particularly massive disturbance occurred in the past at the local soil sampling site. A similar behavior was often observed in soil samples from archaeological excavations or very special sampling sites. Sample HB58-2 was taken from filling material from a prospection shaft of pre-industrial coal mine near Bennstedt. The different characteristics of the filling material and the surrounding soil material were clearly shown by archaeological findings confirming a translocation of soil material [33]. Sample B76 was taken from the entrance area of a karst cave near Bad Frankenhausen. Here, in the 1950s, a Bronze-Age cave sanctuary was investigated by archaeological excavations [34]. The sample HB62-1 originated from an archaeological cut of an ash deposit from a pre-industrial saline place (Bad Dürrenberg). It is marked by a particularly high electrical conductivity due to the high salt content preserved up to now in the related soil layer [35]. HB4 comes from a place in the castle area of the city of Altenburg (Germany). The sample was taken because a non-ferrous metal handyman worked at this place in the Middle Ages. Among other bacteria, the sample supplied a strain of Rhodococcus erythropolis with an exceptionally high tolerance to cobalt [36]. 

In principle, massive disturbances and local conversion and translocation of soil material can not only caused by human activities but could also be the result of growing roots, animal burrows, local erosion, or earthquakes, for example. However, such disturbances can be identified by archaeologists, in principle, during their investigation of the sampling site and its nearby local environment.

**Figure 8:**
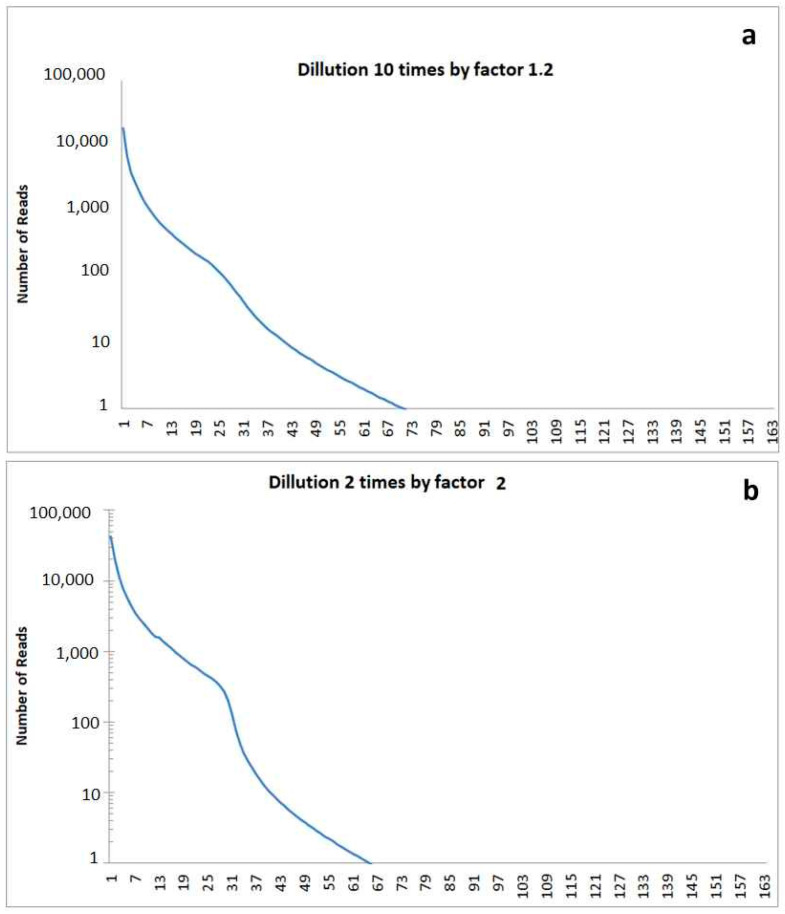
Simulated rank functions in case of disturbance of soil in the past resulting in a dilution of cells: (**a**) cell dilution modelled by ten small dilution steps of factor 1.2 resulting in a weak shoulder in the abundance rank distribution; (**b**) cell dilution modelled by two high dilution steps of factor 2 resulting in a high shoulder in the abundance rank distribution.

For modelling, a dilution event was simulated, in which the concentration of all the bacteria present was reduced. After this event, new species or bacteria evolved. The resulting model rank order functions are marked by a shoulder. The height of this shoulder depends on the dilution factor for the bacterial community existing before the disturbance. The position of the shoulder in the rank order function depends on the timespan since the disturbance. A shoulder at higher abundances indicates a comparatively recent disturbance, and a shoulder at lower values in the rank order function indicates an event in the distant past (Figure 8).

The fitting of the exponential decay in the rank diagram for mediate, resp. lower-abundance OTUs is marked by correlation coefficients (logarithmic values) between experimental and calculated values above 0.99 in most cases (Supplementary Table S1). Only in the case of sample HB4 is the correlation much worse (0.954). This can be explained by the special character of this sample, displaying a very strong shoulder and a low total number of OTUs. All other samples, including the samples with weak and even those with a strong shoulder, are marked by high correlation coefficient over a considerable range of abundances and ranks.

Given how recent our investigations are, it is difficult to differentiate between natural disturbances and ancient human impact using NGS data only. However, such differences can be discussed by reconsidering additional information about the soil samples and their local surroundings as they relate to the archaeological situation and findings.

Despite the fact that rank orders have been used in evolutionary biology and ecology for a long time, most studies have focused—to the best of our knowledge—on finding suitable smooth fitting functions for abundance distributions, Examples of such approaches are made for abundances on the level of multicellular organisms such as groups of forest spiders [14] as well as on the level of genes for the evaluation of the fitness of gene variants [37]. As far as we can see, rank functions of soil bacterial abundances have neither been applied for comparing bacterial communities from archaeological samples nor for developing multi-phase models and detecting disturbances in the evolution of soil microbial communities caused by former impacts. Our results suggest that the combination of fitting abundance distribution functions with analysis of local deviations (steps, shoulders) from fitted rank functions allows for a fast comparison of soil samples and can indicate specific differences in the fates of soil layers and support the interpretation of archaeological findings. 

## 4. Conclusions

The investigations show that the shape of rank functions of abundances in soil bacterial communities could be interpreted by a temporal order of a significant majority of bacterial types in the community. A certain “standard rank order function” can be quantitatively described by the superposition of a simple exponential and an enforced exponential decay. It is hypothesised that both of these decay processes are related to the fast decay of active cells and the slow decay of less active or completely dormant forms of cells. The difference in the decay functions of the assumed both groups of cells could be explained by the higher sensitivity of the active cells and the high robustness of the dormant cells. Deviations from this standard rank order function can be interpreted as massive disturbances of the soil in the past, for example, by translocation of soil material by former human impacts. Thus, the often-observed shoulders in the rank functions of soil bacterial abundances of samples from archaeological excavations could be an indicator of massive disturbances in local environmental conditions in soil in the past.

The results suggest that the investigation of soil microbial communities from archaeological samples and, in particular, an analysis of quantitative relations reflected by rank orders, can contribute to the interpretation of archaeological findings. In addition, such an approach can help to come to a better understanding of evolution of bacterial communities in buried soils and of the consequences of different ancient human impacts, explaining how translocation of soil material in the past affects ecological conditions in recent soil. 

Alongside the possibility of fast rough evaluation and comparison of soil bacterial communities using rank functions, we assume that there is an enormous potential to extract archaeologically relevant information from soil microbial genetic data, which has not been tapped up to now. Future metagenomics studies could supply detailed pictures of the presence and quantitative relations between soil microbes in former human-impacted buried layers, including information about former local ecological situations and the metabolic functions of single species and metabolic networks. In addition, it could become possible to reconstruct ancestry lineages of single bacterial strains and use them for identifying relations between different archaeological sites and related human-associated plants, animals, and eukaryotic microorganisms. 

Despite applications for archaeological samples, the approach presented here might shed light on the general role of translocation of soil material and related local changes in environmental conditions. On the one hand, former surfaces are covered, and the growth processes of most microorganisms in buried former topsoil layers are changed, mostly likely being slowed down. On the other hand, the relocation of soil material from deeper layers brings the residues of ancient communities to the surface and gives dormant bacteria the chance to become active and to multiply. This can occur by natural processes (for example, by erosion) as well as human activities (for example, by mining). Thus, the translocation of soil material means an ecological coupling between former and recent microbial communities. Depending on the time periods between the former deposition and covering of buried layers and their reactivation, such couplings can concern the bridging of historical or prehistorical as well as geological timespans. Strains of microbes which were taken out from active ecosystems in the past can be re-introduced into ecological interactions by such soil translocations. It is possible that abundance rank functions can provide a first rough picture and motivate more detailed studies on ecological couplings bridging timescales over years, centuries, and even geological eras.

## Figures and Tables

**Table 1:** Soil samples.

Intern. Lab-Code	Place	GK-Coordinates	Sample Character
B32	Katzhütte	4,434,441/5,600,633	silicate rock-based forest soil
B43	Sondershausen	4,418,896/5,690,400	shell limestone forest soil, prehistoric rampart
B47	Hachelbich	4,428,625/5,690,096	soil of arable land
B76	Bad Frankenhausen	4,435,851/5,693,032	limestone cave, prehistoric cult place
E64	Welfesholz	4,467,256/5,722,008	pre-industrial copper mining area
E66	Hettstedt	4,467,304/5,722,211	pre-industrial copper mining area
HB4	Altenburg	4,531,000/5,650,500	Medieval non-ferrous metal forge
HB22-1	Großengottern	about 4400/5669	archaeological excavation/bronze ring
HB36-1	Jena	4,471,400/5,643,900	pre-industrial tannery area
HB58-2	Bennstedt	4,488,789/5,706,398	pre-industrial coal prospection shaft
HB62-1	Bad Dürrenberg	4,504,487/5,685,134	pre-industrial saline ash deposit place
T92	Bad Kösen	4,479,712/5,663,779	forest soil near a Middle-Age Castle

## Data Availability

The original contributions presented in this study are included in the article/Supplementary Material. Further inquiries can be directed to the corresponding author(s).

## Supplementary Materials

The following supporting information can be downloaded at: https://www.mdpi.com/article/10.3390/microorganisms12112243/s1, Table S1: Correlation coefficients for the decay in the logarithmic range of rank diagram (mediate and/or low abundant OTUs) (arbitrarily chosen range related to the total number of OTUs (one or more reads) and the appearance of shoulders).
