# Supplementary material for: Soil Bacteria in Archaeology: What Could Rank Abundance Functions Tell Us About Ancient Human Impacts on Microbial Communities?
Source: Microorganisms. 2024 Nov 6;12(11):2243. doi: 10.3390/microorganisms12112243 (PMC11596836; doi:10.3390/microorganisms12112243)
Supplement: Supplementary file 1 [file microorganisms-12-02243-s001.zip › microorganisms-3252444-supplementary.pdf]

Supplementary Table S1

Correlation coefficients for the decay in the logarithmic range of rank diagram (mediate and/or low abundant OTUs)

(arbitrarily chosen range related to the total number of OTUs (one or more reads) and the appearance of shoulders)

Sample	Number of OTUs	Fitting range (No)	Correlation Coefficient	Character
B32	407	170-270	0.9903	weak shoulder
B43	763	20-600	0.9966	no shoulder
B47	733	150-550	0.9977	no shoulder
B76	235	60-80	0.9936	strong shoulder
E64	669	150-400	0.9981	no shoulder
E66	485	220-420	0.9938	weak shoulder
HB4	92	17-25	0.9540	strong shoulder
HB22-1	289	120-200	0.9872	weak shoulder
HB36-1	601	200-400	0.9917	weak shoulder
HB58-2	279	70-135	0.9946	strong shoulder
HB62-1	355	50-200	0.9980	moderate shoulder
T92	761	200-560	0.9944	no shoulder
